# Emergence of Eurasian Avian-Like Swine Influenza A (H1N1) virus in a child in Shandong Province, China

**DOI:** 10.1186/s12879-024-09441-7

**Published:** 2024-06-01

**Authors:** Yujie He, Shaoxia Song, Jie Wu, Julong Wu, Lifang Zhang, Lin Sun, Zhong Li, Xianjun Wang, Zengqiang Kou, Ti Liu

**Affiliations:** 1 Shandong Provincial Center for Disease Prevention and Control, Jinan, China; 2 Binzhou Center for Disease Prevention and Control, Binzhou, China

**Keywords:** Influenza A virus, EAS-H1N1, Molecular characteristics, Phylogenetic analysis, Triple-reassortant

## Abstract

**Background:**

Influenza A virus infections can occur in multiple species. Eurasian avian-like swine influenza A (H1N1) viruses (EAS-H1N1) are predominant in swine and occasionally infect humans. A Eurasian avian-like swine influenza A (H1N1) virus was isolated from a boy who was suffering from fever; this strain was designated A/Shandong-binzhou/01/2021 (H1N1). The aims of this study were to investigate the characteristics of this virus and to draw attention to the need for surveillance of influenza virus infection in swine and humans.

**Methods:**

Throat-swab specimens were collected and subjected to real-time fluorescent quantitative polymerase chain reaction (RT‒PCR). Positive clinical specimens were inoculated onto Madin-Darby canine kidney (MDCK) cells to isolate the virus, which was confirmed by a haemagglutination assay. Then, whole-genome sequencing was carried out using an Illumina MiSeq platform, and phylogenetic analysis was performed with MEGA X software.

**Results:**

RT‒PCR revealed that the throat-swab specimens were positive for EAS-H1N1, and the virus was subsequently successfully isolated from MDCK cells; this strain was named A/Shandong-binzhou/01/2021 (H1N1). Whole-genome sequencing and phylogenetic analysis revealed that A/Shandong-binzhou/01/2021 (H1N1) is a novel triple-reassortant EAS-H1N1 lineage that contains gene segments from EAS-H1N1 (HA and NA), triple-reassortant swine influenza H1N2 virus (NS) and A(H1N1) pdm09 viruses (PB2, PB1, PA, NP and MP).

**Conclusions:**

The isolation and analysis of the A/Shandong-binzhou/01/2021 (H1N1) virus provide further evidence that EAS-H1N1 poses a threat to human health, and greater attention should be given to the surveillance of influenza virus infections in swine and humans.

## Background

Influenza viruses can be divided into four classes, A, B, C and D, according to differences in the viral nucleoproteins and matrix proteins. Influenza A viruses can be further divided into a number of subtypes according to the protein structure and genetic characteristics of haemagglutinin (HA) and neuraminidase (NA) [1]. In addition to humans, influenza A virus infection is also common in animals such as poultry and swine. Both α-2,3-linked sialic acids (Sias) (avian influenza virus receptor) and α-2,6-linked Sias (human influenza virus receptor) are found in swine; therefore, swine are recognized as genetic mixing vessels for human and avian influenza viruses, enabling the reassortment of influenza viruses from different species [2, 3]. Swine are intermediate hosts that can produce new types of latent infectious viruses [4].

Swine influenza virus (SIV) occasionally infects humans, and the main subtypes of influenza A virus circulating in swine are H1N1, H3N2 and H1N2. Each subtype includes a variety of different lineages; and H1N1 swine viruses can be classified into the lineages classical swine H1N1 (CS H1N1), Eurasian avian-like H1N1 (EAS-H1N1) or triple-reassortant H1N1 (TR-H1N1) [5–7]. Unlike human influenza viruses, the distribution of SIVs is regional [8]. In 1979, eight H1N1 influenza segments of avian origin were isolated from swine for the first time [9]; the virus subsequently became widespread in swine in Europe and Asia, resulting in the virus being named Eurasian avian-like H1N1 (EAS-H1N1) [10]. In 1986, cases of EAS-H1N1 infection in humans were identified in Switzerland and the Netherlands [11]. SIVs have been prevalent in the Chinese swine population since 2005, and the prevalent virus types in the Chinese swine population are pandemic H1N1 (A(H1N1) pdm09), EAS-H1N1, CS H1N1, and triple-reassortant swine influenza H1N2 (TR-H1N2) [12]. In 2010, SIVs were able to breach the species barrier and infect humans in China [13]; since then, additional zoonotic cases related to EAS-H1N1 infection have been reported in China, indicating the potential to cause a human influenza pandemic. In this study, we isolated and characterized a novel triple-reassortant EAS-H1N1 strain from a two-year-old boy in Shandong Province.

## Methods

### Epidemiologic information, sample collection and virus identification

A two-year-old boy who lived in the countryside with his grandfather and grandmother presented with a fever of 38.4 °C on the morning of January 1, 2021. Ibuprofen, antipyretic suppositories and infantile acetaminophen Huangnamin granules were used as remedies at home but had no effect. The boy’s fever was 39.3 °C at 1 am and 3 am on the morning of January 2, and the highest temperature reached was 40 °C at 9 am. At 2 PM, the boy was admitted to the Department of Pediatrics of Binzhou People’s Hospital for curative treatment. A 2019 novel coronavirus test was subsequently performed, and the results were negative. After intravenous infusion of ceftriaxone, the patient recovered. On the evening of January 2, the boy’s temperature returned to normal, and after 3 days of outpatient infusion, he was considered healthy.

Binzhou People’s Hospital, a national sentinel hospital for the influenza surveillance network in Shandong Province, conducted influenza and avian influenza virus detection in accordance with the requirements of the national and provincial influenza and avian influenza surveillance programs. The collected throat swab specimens were subsequently transferred to the Binzhou Center for Disease Control and Prevention (Binzhou CDC), where they were subjected to real-time fluorescent quantitative polymerase chain reaction (RT–PCR) for influenza virus detection. Nucleic acid was extracted with an RNeasy Mini Kit (Qiagen, Germany), and RT–PCR was performed with FluA; seasonal H1 and H3; avian H5, H7, and H9; and EAS-H1N1 RT–PCR kits (Bojie, Shanghai, China) in accordance with the manufacturer’s instructions. On January 9, throat swabs were collected from all of the patient’s close contacts, and swine nose swabs and environmental specimens were collected for influenza virus detection using the methods described above; however, all the results were negative.

### Virus isolation and antigenic characteristics

The boy’s positive clinical specimen was inoculated onto Madin-Darby canine kidney (MDCK) cells and cultured with serum-free Dulbecco’s modified Eagle medium (DMEM; Gibco, USA) in the presence of 2.0 µg/ml TPCK-treated trypsin (Sigma, USA) at 35 °C in an atmosphere of 5% CO_2_. The cytopathic effect (CPE) was checked every day, and the medium was collected when the CPE was 75–100%. The presence of the virus in culture was confirmed by a haemagglutination assay following standard protocols using a 1% suspension of guinea pig red blood cells according to the manual provided by the Chinese National Influenza Center (CNIC) [14].

### Genome sequencing and phylogenetic analysis

Viral RNA was extracted from 150 µl of virus stock using an RNeasy Mini Kit (Qiagen, Germany) according to the manufacturer’s instructions. Then, RT‒PCR was performed to determine the viral type, and reverse transcription and whole-genome capture were performed with an influenza A virus whole-genome capture kit (MicroFuture, Beijing, China). Whole-genome sequencing was carried out using an Illumina MiSeq platform (Illumina, USA). Sequences were edited using the CLC Genomics Workbench (Qiagen, Germany). Sequence alignment and phylogenetic analysis were performed with MEGA X software (The Biodesign Institute, USA), and neighbour-joining trees were assembled with bootstrap values determined from 1000 replicates. All reference sequences were downloaded from the EpiFlu database of the Global Initiative on Sharing All Influenza Data (GISAID). The key molecular features were observed and analysed by using Megalign from DNAStar (Madison, WI, USA) through alignment with other representative reference viruses, including A/California/07/2009 (pandemic H1N1), A/swine/Guangdong/1/2010 (swine infection with EAS-H1N1), A/Jiangsu/1/2011, A/Hebei-Yuhua/SWL1250/2012, A/Hunan/42443/2015, and A/Fujian-cangshan/SWL624/2016 (human infection with EAS-H1N1). Genomic glycosylation was analysed using a network glycosylation prediction tool (http://www.cbs.dtu.dk/services/NetNGly).

## Results

### Patient and epidemiology survey

A previously healthy two-year-old boy with no history of travel presented with influenza-like illness (ILI) symptoms on January 1, 2021, and a fever of 38.4 °C. He recovered within one week after receiving an outpatient infusion. A retrospective investigation was conducted to identify the potential source of infection and any other possible cases. The patient lived with his grandfather and grandmother. He had no contact with his neighbours before the symptoms started; his family had two dogs and no other animals, while their neighbours raised swine in captivity. However, the patient’s cousin who already had cold symptoms visited the patient’s home before the patient’s symptoms started. Moreover, the grandson of the patient’s cousin experienced cold symptoms in late December. However, none of the people who were in close contact with the patient developed ILI symptoms during the period of the investigation, and the RT–PCR results of throat swabs were negative.

### Sample identification and viral isolation

The first throat-swab specimen was sent to the Binzhou CDC influenza surveillance network laboratory. RT‒PCR revealed that the sample was positive for influenza A virus but negative for H1N1 pdm09, H3N2, H5, H7, and H9 influenza viruses and positive for EAS-H1N1 influenza virus. The virus was isolated in MDCK cells and designated A/Shandong-binzhou/01/2021 (H1N1) (SD/01/2021). All specimens taken from the patient’s close contacts, the neighbours’ swine and the environment were negative for influenza virus.

### Molecular characteristics

Full-length sequences of the 8 gene segments of A/Shandong-binzhou/01/2021 (H1N1) (PB2, PB1, PA, HA, NP, NA, MP and NS) were obtained, consisting of 2280, 2274, 2151, 1701, 1515, 1410, 982 and 838 nucleotides, respectively (only the nucleotides in the open reading frame were counted). The sequences of all eight segments were submitted to GISAID (accession numbers: EPI2608168- EPI2608175).

**Fig. 1:**
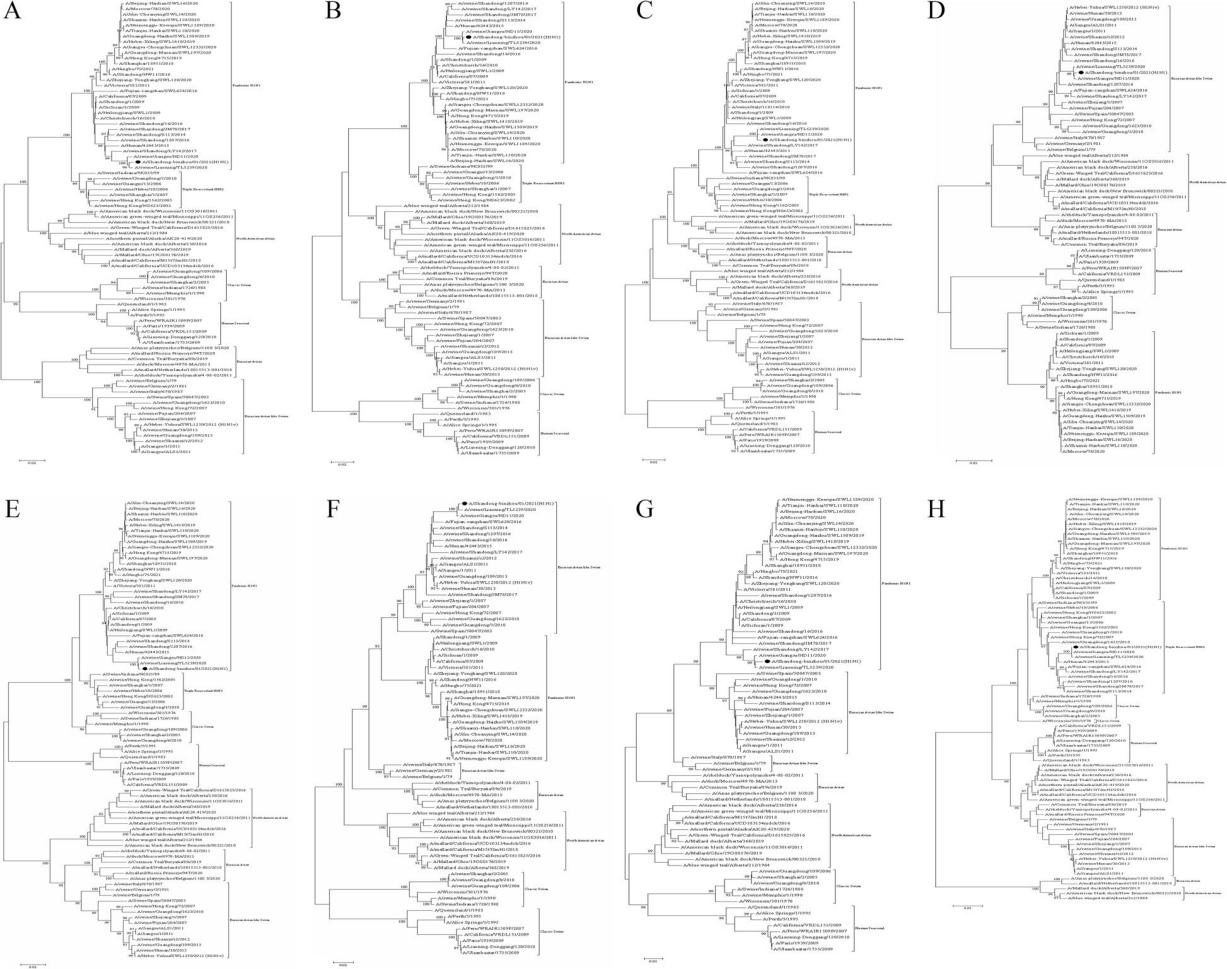
Phylogenetic analysis of the A/Shandong-binzhou/01/2021 (H1N1) gene segments PB2 (**A**), PB1 (**B**), PA (**C**), HA (**D**), NP (**E**), NA (**F**), MP (**G**), and NS (**H**). The reliability of the trees was assessed via bootstrap analysis with 1000 replications. Pandemic H1N1 refers to A(H1N1) pdm09

Phylogenetic analysis revealed that A/Shandong-binzhou/01/2021 (H1N1) was a novel EAS-H1N1 virus containing genes from the Eurasian avian-like swine H1N1 (HA and NA), A (H1N1) pdm09 (PB2, PB1, PA, NP and MP), and triple-reassortant swine influenza H1N2 (NS) strains (Fig. 1; Table 1). All eight segments shared 94.4–97.7% and 94.5–97.0% nucleotide identity with A/Hunan/42443/2015 and A/Fujian-Cangshan/SWL624/2016, respectively (Table 2), and 95.0-99.2% and 94.4–99.2% amino acid identities, respectively (Table 3).

The key molecular features of A/Shandong-binzhou/01/2021 (H1N1) that are known to be associated with increased virulence in mammals, mammalian transmissibility and antiviral susceptibility are shown in Table 4. A/Shandong-binzhou/01/2021 (H1N1) contained the amino acid motif PSIQSR↓GL at the HA1/HA2 cleavage site, which suggested that the virus was a low pathogenic influenza virus. The glycosylation prediction of the HA protein indicated that there were seven potential glycosylation sites (PGSs) (N-X-S/T): ^27^NNS^29^, ^28^NST^30^, ^40^NVT^42^, ^212^NHT^214^, ^291^NCT^293^, ^498^NGT^500^, and ^557^NGS^559^. The presence of amino acids 190D and 225E in HA suggested that it was easier for A/Shandong-binzhou/01/2021(H1N1) to bind to α-2,6-linked Sias in swine and humans.

The amino acid substitutions H274Y and N294S, which are associated with reduced susceptibility to NA inhibitors, were not observed, suggesting that the isolated virus was sensitive to the antiviral drugs oseltamivir and zanamivir. The glycosylation prediction of the A/Shandong-binzhou/01/2021(H1N1) NA protein indicated that there were six potential glycosylation sites (N-X-S/T): ^44^NQS^46^, ^58^NNT^60^, ^63^NQT^65^, ^68^NVS^70^, ^146^NGT^148^, and ^235^NGS^237^.

The MP sequence of A/Shandong-binzhou/01/2021(H1N1) was 982 bp in length with two partially overlapping coding regions. 1 ~ 759 nt, encoding the M1 protein, and 1 ~ 26 nt and 715 ~ 982 nt, encoding the M2 protein. The S31N mutation, which occurred in the M2 protein as observed in A/California/07/2009(H1N1), indicated that the virus had developed resistance to amantadine and rimantadine.

In the PB1 polymerase segment, A/Shandong-binzhou/01/2021(H1N1) contained the 99 H mutation, and the a + 1 alternate open reading frame (ORF) at 209 ~ 367 nt encoded a PB1-F2 truncated peptide of 52 aa. In addition, several amino acid substitutions related to virus virulence or host adaptation have been reported, including L89V, R251K, T271A, and Q591R in PB2 polymerase; L336M and K356R in PA; Q357K in NP; and P42S in NS1. A/Shandong-binzhou/01/2021(H1N1) gene sequences included all these amino acid substitutions.

**Table 1:** Genetic origin of A/Shandong-binzhou/01/2021(H1N1) virus isolated from a human in Shandong, China

Isolates	Lineage assigned to gene segment							
	PB2	PB1	PA	HA	NP	NA	MP	NS
A/California/07/2009(H1N1)	PDM	PDM	PDM	PDM	PDM	PDM	PDM	PDM
A/swine/Guangdong/1/2010^b^	TR	TR	TR	EAS	TR	EAS	EAS	TR
A/Jiangsu/1/2011^a^	EAS	EAS	EAS	EAS	EAS	EAS	EAS	EAS
A/Hebei-Yuhua/SWL1250/2012^a^	EAS	EAS	EAS	EAS	EAS	EAS	EAS	EAS
A/Hunan/42443/2015^a^	PDM	PDM	PDM	EAS	PDM	EAS	EAS	TR
A/Fujian-cangshan/SWL624/2016^a^	PDM	PDM	PDM	EAS	PDM	EAS	PDM	TR
A/Shandong-binzhou/01/2021(H1N1)	PDM	PDM	PDM	EAS	PDM	EAS	PDM	TR

PDM, genes with the closest homology to A(H1N1) pdm09 viruses

EAS, genes with the closest homology to Eurasian avian-like swine influenza viruses

TR, genes with the closest homology to triple-reassortant swine influenza H1N2 virus

^a^ Human infection with Eurasian avian-like swine influenza virus

^b^ Swine infection with Eurasian avian-like swine influenza virus

**Table 2:** Nucleotide homology analysis of the eight gene segments of A/Shandong-binzhou/01/2021 (H1N1)

Isolates	A/Shandong-binzhou/01/2021(H1N1) (nucleotide identities %)							
	PB2	PB1	PA	HA	NP	NA	MP	NS
A/California/07/2009(H1N1)	96.5	97.4	96.4	73.7	97.1	88.5	97.5	90.8
A/swine/Guangdong/1/2010^b^	92.7	93.4	92.3	92.4	92.9	90.9	94.7	94.9
A/Jiangsu/1/2011^a^	82.6	84.0	84.2	96.7	82.3	96.0	94.6	82.1
A/Hebei-Yuhua/SWL1250/2012^a^	82.2	84.3	84.1	96.3	82.6	95.6	94.5	81.8
A/Hunan/42443/2015^a^	96.8	97.7	96.6	97.1	97.1	95.9	94.4	97.1
A/Fujian-cangshan/SWL624/2016^a^	95.3	97.0	94.5	95.7	95.7	96.5	96.5	96.5

^a^ Human infection with Eurasian avian-like swine influenza virus

^b^ Swine infection with Eurasian avian-like swine influenza virus

**Table 3:** Amino acid analysis of A/Shandong-binzhou/01/2021 (H1N1) proteins

Isolates	A/Shandong-binzhou/01/2021(H1N1) (identities %)										
	PB2	PB1	PB1-F2	PA	HA	NP	NA	M1	M2	NS1	NS2
A/California/07/2009(H1N1)	97.9	98.3	98.1	98.7	77.8	97.8	90.2	99.6	98.0	87.8	89.4
A/swine/Guangdong/1/2010^b^	95.4	96.8	95.2	96.4	91.7	97.0	91.5	99.2	94.9	91.9	91.1
A/Jiangsu/1/2011^a^	93.2	94.2	66.7	93.3	96.3	92.4	95.1	98.4	94.9	79.6	89.4
A/Hebei-Yuhua/SWL1250/2012^a^	93.0	94.3	64.8	93.5	96.1	92.2	94.7	98.4	94.9	78.3	87.8
A/Hunan/42443/2015^a^	97.9	98.2	98.1	99.2	96.3	97.4	95.8	98.0	96.0	95.0	95.1
A/Fujian-cangshan/SWL624/2016^a^	96.8	98.0	94.4	97.4	95.6	97.6	96.8	99.2	97.0	95.0	95.9

^a^ Human infection with Eurasian avian-like swine influenza virus

^b^ Swine infection with Eurasian avian-like swine influenza virus

**Table 4:** Molecular analysis of A/Shandong-binzhou/01/2021 (H1N1) compared to other viruses

Gene product	Function	Amino acid substitution	Virus^c^							References
			CA	GD	JS	HB	HN	FJ	SD	
HA^a^	Antigenic drift	G158E	G	**E**	G	G	G	G	G	[15]
	Easy to bind to human receptors	E190D	**D**	V	**D**	**D**	**D**	**D**	**D**	[16]
	Increased efficiency of virus replication	D225E	D	**E**	**E**	**E**	**E**	**E**	**E**	[17]
NA^b^	Resistance to NA inhibitors	H274Y	H	H	H	H	H	H	H	[18, 19]
		N294S	N	N	N	N	N	N	N	[18, 19]
PB2	Enhanced virus adaptability in mice	L89V	**V**	**V**	**V**	**V**	**V**	**V**	**V**	[20]
		R251K	R	R	R	R	R	R	**K**	[21]
		T271A	**A**	**A**	T	T	**A**	**A**	**A**	[22]
		Q591R	**R**	**R**	Q	Q	**R**	Q	**R**	[23]
		E627K	E	E	E	E	E	E	E	[24, 25]
		D701N	D	D	**N**	**N**	D	D	D	[26]
PB1	Between-species transmission	X99H	**H**	**H**	**H**	**H**	**H**	**H**	**H**	[25]
PA	Resistance to baloxavir	E23G/K	E	E	E	E	E	E	E	[27]
	Resistance to baloxavir•	I38T/M/F	I	I	I	I	I	I	I	[28]
	Enhance viral polymerase activity	L336M	**M**	L	L	L	**M**	**M**	**M**	[29]
	Enhance viral polymerase activity, increase virulence in mice	K356R	**R**	K	K	K	**R**	**R**	**R**	[30]
NP	Alter virulence in mice	Q357K	**K**	**K**	Q	Q	**K**	**K**	**K**	[31]
M1	Increase virulence in mice	T215A	**A**	**A**	**A**	**A**	**A**	**A**	**A**	[25, 32]
M2	Resistance to amantadine	S31N	**N**	**N**	**N**	**N**	**N**	**N**	**N**	[33]
NS1	Increase virulence in mice	P42S	**S**	**S**	**S**	**S**	**S**	**S**	**S**	[34, 35]

For each virus, the amino acid positions carrying the substitution are highlighted

^a^ The H3 numbering system was used

^b^ The N2 numbering system was used

^c^ CA, A/California/07/2009(H1N1); GD, A/swine/Guangdong/1/2010; JS, A/Jiangsu/1/2011; HB, A/Hebei-Yuhua/SWL1250/2012; HN, A/Hunan/42443/2015;FJ, A/Fujian-cangshan/SWL624/2016; SD, A/Shandong-binzhou/01/2021 (H1N1)

## Discussion

The EAS-H1N1 virus has emerged and is widely circulating in Chinese swine populations, and there are multiple reports of different genotypes of this virus infecting humans. In 2010, an infection was reported in a three-year-old boy in Jiangsu Province with a history of renal disease who subsequently died. The A/Jiangsu/1/2011 virus isolated in this case showed 99.1-99.8% homology with the EAS-H1N1 virus infecting swine in Jiangsu Province during 2010–2011, indicating that the transmission of the EAS-H1N1 virus in China was a regional epidemic [13]. In 2012, a 3-year-old boy in Hebei Province was diagnosed with EAS-H1N1 virus infection, which was also the first case detected by an influenza-like case surveillance system. The A/Hebei-Yuhua/SWL1250/2012 virus was isolated in this case and showed 98.9-99.6% homology with A/Jiangsu/1/2011 [36]. In 2015, a recombinant EAS-H1N1 virus was detected in Hunan Province; this virus was identified as A/Hunan/42443/2015, and the recombinant mode is shown in Table 2. This recombinant EAS-H1N1 virus caused more pronounced clinical symptoms (with complications such as severe pneumonia, respiratory failure, acute respiratory distress syndrome, and heart failure) in the infected child and greater infectivity and virulence than A/Jiangsu/1/2011 in a mouse model; moreover, the replication of the A/Hunan/42443/2015 virus was substantially greater than that of the A/Jiangsu/1/2011 virus in the respiratory tract of mice [37]. In a BALB/c mouse model, A/Hunan/42443/2015 caused more severe morbidity and higher mortality than North American variant H1 virus isolates. Furthermore, A/Hunan/42443/2015 efficiently replicated throughout the respiratory tract of ferrets and exhibited the capacity for transmission from ferrets [38]. On October 19, 2016, a 46-year-old male in Fujian Province was infected with EAS-H1N1 virus and died of multiple organ failure on October 28. The patient’s throat swab samples were sequenced, and the results revealed that the virus, named A/Fujian-cangshan/SWL624/2016, was a triple-reassortant virus that contained gene segments from the EAS-H1N1 (HA and NA), triple-reassortant swine influenza H1N2 (NS) and A (H1N1) pdm09 (PB2, PB1, PA, NP and MP) viruses. This genotype of A/Fujian-cangshan/SWL624/2016 showed the potential to spread in humans and developed a degree of drug resistance that facilitated its spread [24]. In 2018, in Tianjin, an EAS-H1N1 virus, named A/Tianjin-baodi/1606/2018 (H1N1), was isolated from a boy with obvious influenza symptoms. The recombinant mode of A/Tianjin-baodi/1606/2018(H1N1) was similar to that of A/Fujian-cangshan/SWL624/2016, which demonstrated similar strong transregional transmissibility to that of EAS-H1N1 [25]. In 2020, a one-year-old girl in Yunnan Province was diagnosed with EAS-H1N1 infection via an influenza-like case surveillance system. Phylogenetic analysis of this isolated virus, named A/Yunnan-Mengzi/1462/2020(H1N1v), revealed similar results to those of A/Hunan/42443/2015, and the HA and NA segments shared 97.9% and 97.4% nucleotide identity, respectively, with A/Hunan/42443/2015 [39]. All these cases indicate that EAS-H1N1 viruses continue to circulate in swine and accumulate mutations and recombinations, yielding reassorted viruses that occasionally infect humans.

Haemagglutinin (HA) is a trimeric glycoprotein that is present in multiple copies in the membrane envelope of influenza virus [40–42]. Wang et al. generated an HA mutant by reverse genetics, and the amino acid glutamic acid (E) at position 225 in the HA domain of the EAS-H1N1 virus was found to accelerate the replication of the virus in guinea pigs. This study indicated that amino acid 225E in HA plays a key role in EAS-H1N1 virus transmission [17]. In addition, the HA domain of the EAS-H1N1 virus circulating in China also contains an E190D mutation. A virus harbouring the 190D mutation more easily binds to the α-2,6-linked Sias receptor and is thus able to be transmitted within the population [16]. A/Shandong-binzhou/01/2021 (H1N1) contains both mutation sites, 225E and 190D, within the HA protein, which indicates that the virus may preferentially bind to human upper respiratory tract receptors.

Influenza viruses escape antibody-mediated neutralization by continually changing the amino acid residues in their HA head domain, which induces antigenic drift. Persistent antigenic drift encourages influenza viruses to evade the host immune response, strongly affecting the efficacy of vaccines. Wang et al. revealed that the G158E mutation in the HA protein could reduce the affinity of the HA protein for antibodies, and the spatial structure of the HA protein also changes due to this mutation, which promotes both the antigenic shift of the EAS-H1N1 virus and its transmission in pigs and human populations [15].

The NA(neuraminidase)-H274Y mutant, which contains a compensating mutation, increases the fitness and transmissibility of influenza viruses, and the NA-N294S mutation may increase the resistance of viruses to NA inhibitors (NAIs) [18, 19]. The NA-H274 and NA-N294 in A/Shandong-binzhou/01/2021 (H1N1) indicated that the virus was still sensitive to neuraminidase inhibitors. Therefore, at the early stage of infection, the administration of oseltamivir or zanamivir may reduce the severity of infection.

The PB2-Q591R, E627K and D701N mutant have been demonstrated to be critical for increasing polymerase activity, and enhancing viral replication and transmissibility in mammalian models [23–26]. In addition, related studies revealed that the PB2-L89V, R251K and T271K mutation could increase virulence and virus adaptability in mammalian [20–22]. Although the A/Shandong-binzhou/01/2021 (H1N1) virus PB2 gene possesses amino acid mutations 89V, 251K, 271K and 591R, the virus strain lacks characteristic human/mammalian amino acid mutations to enhance transmissibility in mammalian, such as E627K and D701N.

The NP protein stands out as a high-profile drug target against the background of increasing viral resistance, and the NP-Q357K substitution of the EAS-H1N1 viruses alter the virulence phenotype. The NP-Q357K substitution is prevalent in human viruses and rarely detected in avian influenza viruses, but the fact that this substitution readily occurs when avian influenza viruses circulate in pigs and may facilitate their infection of humans should receive more attention [31].

PB1-F2 is an accessory protein that is encoded by an a + 1 alternate open reading frame (ORF) in the PB1 segment. The length of the PB1-F2 peptide, which is longer than 79 aa, is an important factor affecting the toxicity of PB1 through its apoptosis-inducing activity, and amino acids 46–75 of the PB1-F2 peptide are important for the localization and targeting of mitochondria [43]. The truncated peptide, 52 aa in length, produced by A/Shandong-binzhou/01/2021 (H1N1) may lack the ability to induce apoptosis through the mitochondrial pathway.

The PA endonuclease catalytic site has been regarded as an attractive target for screening anti-influenza drugs [44]. Recent studies have revealed that the I38T/M/F substitution in PA could reduce susceptibility to the active metabolite baloxavir (BXM) [28]. In addition, studies suggest that E23G/K, alone or combined with I38T, is another important marker that can reduce BXM susceptibility [27]. Although residue I of the A/Shandong-binzhou/01/2021 (H1N1) PA protein is the same as that of other EAS-H1N1 viruses mentioned here, mutants may emerge and/or be transmitted among humans; thus, this hotspot for substitution should be monitored. According to related research, the PA-336 M mutation significantly enhanced pathogenicity in a mouse model [29], and the PA-K356R mutation in human A549 cells increased the nuclear accumulation of PA and increased viral polymerase activity. The PA-K356R mutant in mice also enhanced virus replication and caused lethal infection [30]. The PA-336 M and PA-356R mutations in A/Shandong-binzhou/01/2021 (H1N1) suggest that additional research on the pathogenesis of this virus in mice should be performed in the future, and additional attention should be given to this virus.

Influenza virus matrix protein (MP) encodes two viral proteins, matrix protein M1 and membrane protein M2 [45, 46]. Research has revealed that the M1-T215A mutation significantly enhances EAS-H1N1 virulence in mice [25, 32], and A/Shandong-binzhou/01/2021 (H1N1), similar to other EAS-H1N1 viruses isolated from humans, exhibited antiviral resistance to amantadine and rimantadine caused by the M2-S31N mutation blocking the M2 proton channel and preventing viral uncoating [33]. The 42S mutation of the nonstructural protein NS1 in the EAS-H1N1 virus can regulate the host’s IFN response by blocking the activation of IRF3, which results in high levels of IFN-α and IFN-β production and facilitates virus replication [34, 35], the NS1-42S mutation in A/Shandong-binzhou/01/2021 (H1N1) indicated that the virus may be more virulent.

In conclusion, EAS-H1N1 viruses occasionally infect humans. We report an EAS-H1N1 reassortant virus, A/Shandong-binzhou/01/2021 (H1N1), which was isolated from a boy in Shandong Province with an influenza-like phenotype. The virus contained 2 surface genes from the EAS-H1N1 virus, 5 internal genes from the A(H1N1) pdm09 virus, and 1 internal gene from the triple-reassortant swine influenza H1N2 virus. Whole-genome sequencing revealed a number of gene mutations encoding amino acid substitutions that have been associated with increased virulence and the likelihood of transmission to other mammals and humans. Therefore, continuing and increasing surveillance of influenza viruses in swine is highly important because they represent a host species that can produce novel viruses that may lead to another human pandemic.

## Data Availability

All data generated or analysed during the study are included in this article. More data on the gene sequences are available at GISAID EpiFlu under the isolate ID:EPI_ISL_17859365 with the accession numbers EPI2608168-EPI2608175.

## Abbreviations

EAS-H1N1: Eurasian avian-like swine influenza A (H1N1)
RT‒PCR: Real-time fluorescent quantitative polymerase chain reaction
MDCK: Madin–Darby canine kidney
HA: Haemagglutinin
NA: Neuraminidase
NP: Nucleoprotein
PB2: Polymerase basic 2
PB1: Polymerase basic 1
PA: Polymerase acidic
MP: Matrix protein
NS: Nonstructural protein
Sias: Sialic acids
SIV: Swine influenza virus
CS H1N1: Classical swine H1N1
TR H1N1: Triple-reassortant H1N1
A(H1N1) pdm09: Pandemic H1N1
TR H1N2: Triple-reassortant swine influenza H1N2
CPE: Cytopathic effect
CNIC: Chinese National Influenza Center
GISAID: Global Initiative on Sharing All Influenza Data
ILI: Influenza-like illness
